# Synergetic Effects of Combined Treatment of Colistin With Meropenem or Amikacin on Carbapenem-Resistant *Klebsiella pneumoniae in vitro*

**DOI:** 10.3389/fcimb.2019.00422

**Published:** 2019-12-10

**Authors:** Lan Yu, Jisheng Zhang, Yanjun Fu, Yongxin Zhao, Yong Wang, Jing Zhao, Yuhang Guo, Chunjiang Li, Xiaoli Zhang

**Affiliations:** ^1^Department of Microbiology, Yongchuan Hospital of Chongqing Medical University, Chongqing, China; ^2^Department of Microbiology, The First Affiliated Hospital of Jiamusi University, Jiamusi, China; ^3^Department of Scientific Research Section, Jiamusi University School of Clinical Medicine, Jiamusi, China; ^4^Department of Pathogenic Biology, Jiamusi University School of Basic Medicine, Jiamusi, China

**Keywords:** carbapenem-resistant *Klebsiella pneumoniae*, colistin, combination therapy, synergistic effect, bactericidal effect

## Abstract

The purpose of this study was to investigate the synergistic and bactericidal effects of combinations of colistin with meropenem or amikacin *in vitro* and provide laboratory data needed for development of therapeutic strategies for the treatment of carbapenem-resistant *Klebsiella pneumoniae* (CRKP) infection. We found that minimum inhibitory concentration (MIC) of colistin, meropenem and amikacin were 2~32, 4~256, and 1~16384 μg/ml, respectively. The minimum bactericidal concentration of the antibiotics was either 1× or 2×MIC. Treatments of 6 CRKP isolates at 1 μg/ml colistin completely killed 2 of them and suppressed 4 others growth. 4 CRKP isolates at 16 μg/ml meropenem or amikacin completely killed and suppressed 2 others growth. 2 CRKP isolates showed synergic effects in all colistin combination and 3 CRKP isolates showed synergic effects in part of colistin combination. Our data suggest that colistin in combination with either meropenem or amikacin could be a valid therapeutic option against colistin-resistant CRKP isolates. Moreover, the combination of colistin-amikacin is less expensive to treat CRKP infections in Eastern Heilongjiang Province.

## Introduction

The mergence and dissemination of carbapenem-resistant *Klebsiella pneumoniae* (CRKP) may poses serious threat to the elderly and sick persons. These organisms are associated with high mortality rates and have the potential to spread widely around the world (Patel et al., 2008; Grundmann et al., 2017; Zhang et al., 2017). It has been reported that the mortality rates can be as high as 40–50% in patients with CRKP infections (Patel et al., 2008). The pathogens often harbor multiple drug-resistant genes that cause the bacteria hetero-resistance to antibiotics in addition to β-bactams (Band et al., 2018, 2019), severely limiting alternative treatment options and increasing therapeutic cost (Huang et al., 2018; Santos and Secoli, 2019). The development of new antimicrobial agents to combat new CRKP isolates is extremely time-consuming, and therapeutic replacement with novel alternative agents may cause potential toxicities. Recently, Band et al. reported that two multidrug-resistant CRKP isolates exhibiting colistin hetero-resistance, a phenomenon in which only a subpopulation of genetically identical bacterial is resistant to the drug (Band et al., 2018, 2019). Therefore, the use of combination therapy of two or more antibiotics instead of monotherapy in CRKP-infected patients is a feasible option, and the efficacy of such combinations to kill the hetero-resistant CRKP isolates remains to be determined.

In our previous studies, we have demonstrated that carbapenem-non-susceptible *Enterobacteriaceae* (CNSE) isolates from the Eastern Heilongjiang Province of China carry carbapenem hydrolases encoded by the blaKPC-2, blaNDM-1, blaNDM-5, blaNDM-7, and blaIMP-4 genes. Most of these isolates harbor multiple drug-resistance genes related to extended-spectrum beta lactamase (ESBL) such as the blaSHV, blaTEM, blaCTX-M-15, and CTX-M-177 genes (Gong et al., 2018). The Carbapenemase-producing *K. pneumoniae* (KP) isolates are sometime dangerous because these pathogens are frequently co-resistant to multiple antibiotic classes, and only a few of available antibiotics remain effective. So far, several combinations such as colistin (COL), tigecycline (TGC), gentamicin (GEN), and fosfomycin (FM) have been reported to be effective against CRKP isolates as measured by *in vitro* checkerboard technique or time-kill assays (Morici et al., 2017)^.^ Clinical retrospective studies have also demonstrated favorable outcomes for the patients treated with combinations of COL and a carbapenem, TGC, FM, or an aminoglycoside (Tangden et al., 2014). Thus, COL has been recommended as the first line of option against CRKP isolates (Gibson et al., 2016). However, effectiveness of COL combinations with meropenem (MEM) or amikacin (AMK) against CRKP isolates emerged in the Heilongjiang Province of China has not been studies yet.

In this study, we investigated the synergistic and bactericidal effects of combinations of COL with either the most sensitive carbapenem antibiotic, MEM or the antibiotic with the lowest resistance rate in our region, AMK against the CRKP isolates resistant to COL or MEM alone. Our studies provided valuable laboratory data of antibiotic susceptibility for clinicians to develop therapeutic strategies for the treatment of drug-resistant CRKP-infections in the eastern Heilongjiang Province, China. In addition, combination therapy may also help to prevent the spread of the bacteria in this region as well as other parts of China.

## Materials and Methods

### Bacterial Isolates

Forty clinical CRKP isolates were collected from patients with infectious diseases at the First Affiliated Hospital of Jiamusi University from October 2015 to January 2019. The CRKP isolates were characterized by using the Vitek 2 system and the AST-GN card (bioMérieux, France). Routine multilocus sequence typing (MLST) and polymerase chain reaction (PCR) were performed to verify the presence of carbapenemase and ESBL genes (Table 1).

**Table 1:** Results for the bla genotype, MLST, MICs, MBCs, and FICIs of colistin, meropenem, and amikacin against CRKP isolates.

**Isolate**	**Source**	**bla genotype**	**MLST**	**MIC (μg/ml)**			**MBC**			**FICI**	
				**COL**	**MEM**	**AMK**	**COL**	**MEM**	**AMK**	**COL+MEM**	**COL+AMK**
700603				4	1/4	1/2					
CRKP1	Sputum	KPC-2, SHV, TEM, CTX-M-15	ST76	8	64	8				0.25	1
CRKP2	Sputum	KPC-2, SHV, TEM, CTX-M-15	ST76	8	128	4				0.25	0.375
CRKP3	Sputum	KPC-2, SHV, TEM, CTX-M-15	ST76	8	128	8				0.25	0.75
CRKP4	Sputum	KPC-2, SHV, TEM, CTX-M-15	ST76	16	16	8				0.375	0.625
CRKP5	Sputum	KPC-2, SHV, TEM, CTX-M-15	ST76	8	16	8				0.375	0.75
CRKP6	Sputum	KPC-2, SHV, TEM, CTX-M-15	ST76	16	16	8				0.375	0.625
CRKP7	Sputum	KPC-2, SHV, TEM, CTX-M-15	ST76	8	16	8				1	0.25
CRKP8	Sputum	KPC-2, SHV, TEM, CTX-M-15	ST76	8	16	4				0.625	0.375
CRKP9	Sputum	KPC-2, SHV, TEM, CTX-M-15	ST76	8	16	4				0.625	0.375
CRKP10	Sputum	KPC-2, SHV, TEM, CTX-M-15	ST76	8	16	8				0.5	0.25
CRKP11	Sputum	KPC-2, SHV, CTX-M-15	ST76	8	16	16				0.5	0.375
CRKP12	Sputum	KPC-2, NDM-5, SHV, TEM, CTX-M-177	ST76	8	64	2	2×	1×	2×	0.375	0.375
CRKP13	Blood	KPC-2, SHV, TEM, CTX-M-15	ST76	4	16	4	1×	1×	1×	0.75	0.25
CRKP14	Blood	KPC-2, SHV, TEM, CTX-M-15	ST76	4	16	2	1×	1×	1×	0.75	0.375
CRKP15	Blood	KPC-2, SHV, TEM, CTX-M-15	ST76	4	64	2	2×	1×	1×	0.375	1
CRKP16	Sputum	KPC-2, SHV, TEM, CTX-M-15	ST76	16	16	8				0.25	0.625
CRKP17	Sputum	KPC-2, SHV, TEM, CTX-M-15	ST76	16	16	8				0.375	0.625
CRKP18	Sputum	KPC-2, SHV, TEM, CTX-M-15	ST76	16	16	16				0.375	0.25
CRKP19	Sputum	KPC-2, SHV, TEM, CTX-M-15	ST76	2	64	2	1×	1×	2×	0.375	0.75
CRKP20	Sputum	KPC-2, SHV, TEM, CTX-M-15	ST76	4	16	2	2×	2×	2×	0.75	0.625
CRKP21	Secretion	KPC-2, SHV, TEM, CTX-M-15	ST76	4	8	4				0.25	0.375
CRKP22	Sputum	KPC-2, SHV, TEM, CTX-M-15	ST76	8	8	8				0.625	0.375
CRKP23	Sputum	KPC-2, SHV, TEM, CTX-M-15	ST76	8	16	4				0.5	0.375
CRKP24	Blood	KPC-2, SHV	ST11	4	128	1	1×	2×	2×	0.625	0.375
CRKP25	Sputum	KPC-2, SHV, TEM, CTX-M-15	ST76	8	16	2	1×	2×	1×	0.5	1
CRKP26	Blood	KPC-2, SHV, TEM, CTX-M-15	ST76	32	32	8				0.25	0.375
CRKP27	Sputum	KPC-2, TEM, CTX-M-15	ST76	8	32	4				0.25	0.375
CRKP28	Sputum	KPC-2, TEM, CTX-M-15	ST76	8	16	4				0.5	0.375
CRKP29	Sputum	KPC-2, SHV, TEM, CTX-M-15	ST76	16	8	4				0.375	0.375
CRKP30	Sputum	KPC-2, SHV, TEM, CTX-M-15	ST76	8	8	4				0.75	0.375
CRKP31	Sputum	SHV, TEM, CTX-M-15	ST76	8	16	4				0.75	0.375
CRKP32	Sputum	KPC-2, SHV, TEM, CTX-M-15	ST375	2	8	1	1×	1×	1×	1	0.625
CRKP33	other specimens	KPC-2, SHV, TEM, CTX-M-15	ST76	8	16	4				0.5	0.5
CRKP34	Sputum	SHV, TEM	ST530	2	4	1	1×	1×	1×	1	0.625
CRKP35	Sputum	SHV, TEM	ST3335	8	4	4				0.75	0.375
CRKP36	Blood	KPC-2, SHV, TEM, CTX-M-177	ST11	8	256	16,384				0.625	–
CRKP37	Sputum	KPC-2, SHV, TEM, CTX-M-15, CTX-M-177	ST290	8	16	2				0.375	0.25
CRKP38	Sputum	KPC-2, SHV, TEM, CTX-M-15, CTX-M-177	ST290	8	16	2				0.375	0.25
CRKP39	Sputum	KPC-2, SHV, TEM, CTX-M-15, CTX-M-177	ST290	8	16	2				0.375	0.25
CRKP40	Sputum	KPC-2, NDM-1, SHV, TEM, CTX-M-15, CTX-M-177	ST290	8	128	1				0.25	0.375

*MLST, multilocus sequence typing; MIC, minimum inhibitory concentrations; MBC, minimum bactericidal concentration; FICI, fractional inhibitory concentration index; COL, colistin; MEM, meropenem; AMK, amikacin; –, not tested*.

### Antimicrobial Susceptibility Testing

Minimum inhibitory concentration (MIC) defined as the lowest compound concentration (μg/ml) required to stop bacterial growth was determined by using the microbroth dilution method according to the Clinical and Laboratory Standard Institutes (CLSI) recommendations (Clinical Laboratory Standards Institute, 2016). An ESBL-positive strain of KP ATCC700603 was used as a reference control. Three antimicrobial agents were tested: Colistin (COL, Sigma-Aldrich, St. Louis, MO), Meropenem (MEM, Haibin Pharmaceutical Co., Shenzhen, China), and amikacin (AMK, Sui Cheng Pharmaceutical Ltd., Tianjin, China). The concentration ranges of COL, MEM and AMK used in this study were 0.0625–64, 0.5–256, and 0.03125–16,384 μg/ml, respectively. The MEM and AMK results were interpreted per CLSI criteria (Clinical Laboratory Standards Institute, 2016), whereas the COL results were interpreted based on the European Committee on Antimicrobial Susceptibility Testing (EUCAST) breakpoint recommendations (EUCAST, 2016). The MICs were determined by measuring optical density (OD) at 570 nm using a microplate reader.

### Bactericidal/Static Determination Assay

After MIC tests, bacteria in each well (100 μL) corresponding to 1×, 2×, 4×, and 8× MIC were transferred and spread on Mueller-Hinton (MH) agar plates and cultured at 37°C overnight, followed by colony-forming units (CFUs) counting. Minimum bactericidal concentrations (MBCs) of the antibiotics were defined as the lowest antibiotic concentrations required to cause ≥99.99% cell death within 24 h. An antibiotic was classified as bactericidal if its MBC was between 1× to 4× MIC.

### Checkerboard Technique

The synergistic effects of COL-MEM and COL-AMK combinations on CRKP isolates were tested using the checkerboard technique. The concentration ranges were determined based on MICs. Briefly, 50 μl of each antibiotic at five increasing (4-fold) concentrations (0.125× to 2× MIC) were used, and each well was inoculated with 100 μl of a 7.5 × 10^5^ CFU/ml suspension of the test CRKP isolates in a final volume of 200 μl in duplicate. Results were measured by reading optical density (OD) after 24 h incubation at 37°C at 570 nm using a microplate reader. The effects of the antimicrobial combinations were defined according to the fractional inhibitory concentration index (FICI) as follows: FICI ≤ 0.5, synergism; 0.5 < FICI ≤ 1, additive; 1 < FICI ≤ 4, indifferent; or FICI > 4, antagonistic (Bai et al., 2015).

### Antibacterial Time-Kill Assay

The antibacterial time-kill assay was performed on three individual antibiotics and two COL combinations against 6 distinct sequence types (STs) of the CRKP isolates. Antibiotic concentrations were calculated using the mean value of the steady-state concentrations of the non-protein-bound drug in humans as described previously (Clinical Laboratory Standards Institute, 1999). The concentrations of 250 and 1,000 μg/L COL, 4,000 and 16,000 μg/L MEM, and 8,000 and 16,000 μg/L AMK were used in both single-agent and combination studies (Kulengowski et al., 2018). Briefly, 5 × 10^5^ CFU/ml of the tested organism were inoculated in 10 ml of the broth containing a single antibiotic or antibiotics combinations. Bacterial growth was quantified after 0, 2, 4, 8, 12, and 24 h incubation at 37°C by plating 10-fold dilutions on sheep blood agar, followed by counting CFU/ml. Time-kill curves were then constructed as a function of time, and the results are represented as the difference in log_10_ between the CFU/ml at 0 and 24 h. A decrease of ≥3 log_10_ in colony number as compared to the initial CFU/ml was considered as bactericidal effect while a decrease of <3 log_10_ CFU/ml over the initial CFU/ml was defined as bacteriostatic effect. An increase in colony count from the previous timepoint was thought as regrowth (Doern, 2014). A decrease of ≥2 log_10_ CFU/ml of combination cultures as compared to the most active single-drug broth at the same timepoint was defined as synergistic effect whereas an increase of >2 log_10_ in the combination cultures was interpreted as antagonism. Additivity and indifference were designated when the outcomes neither meet the criteria of synergy nor the criteria of antagonism (Petersen et al., 2006).

### Statistical Analysis

The Kolmogorov–Smirnov test was used to analyze the data distribution. Student's *t*-test was used for treatment comparisons. SPSS 22.0 software was used for statistical analyses. *P* < 0.05 was considered as statistically significant.

## Results

### Characteristics of Bacterial Isolates

To characterize the isolates, we performed MLST and PCR to exam whether they carry carbapenemase and ESBL genes. We found that out of 40 CRKP isolates, 37 (92.5%) carried KPC-2 gene, which encodes carbapenemase class A, and 2 (13.2%) carried a gene that encodes New Delhi metallo-β-lactamase (NDM) carbapenemase. CRKP40 isolate carried blaNDM-1 gene while CRKP11 isolate harbored blaNDM-5 gene. Among total 6 ESBL genes amplified, blaSHV, bIaTEM, blaCTX-M-15, and blaCTX-M-177 were present in 38 (95.0%), 38 (95.0%), 35 (87.5%), and 6 (15.0%) of the CRKP isolates, respectively. These CRKP isolates were grouped into 6 distinct STs; ST76 was the most predominant clone (*n* = 30), followed by ST290 (*n* = 4), ST11 (*n* = 2), ST375 (*n* = 2), ST570 (*n* = 1), and ST3335 (*n* = 1) as shown in Table 1.

### Bactericidal/Static Determination and Antimicrobial Susceptibility

To determine whether COL, MEM, and AMK prevent bacterial growth or kill bacteria, we carried out bactericidal/static determination assays. Our genotyping and MLST mapping found that 40 strains carried 2–5 different bla genotypes, some of which have the same bla genotyping (Table 1). Thus, we selected 10 CRKP isolates out of 40 for bactericidal/static determination assays because these representative CRKP isolates included all bla genotypes. We found that COL, MEM and AMK were bactericidal since the MBC of COL, MEM or AMK was either 1× MIC or 2× MIC (Table 1). MICs against COL and MEM were ranged from 2 to 32 and 4 to 256 μg/ml, respectively. The CRKP's resistances to single COL and MEM treatment reached 92.5 and 100%, respectively. Of the CRKP isolates, 97.5% were sensitive to AMK. The MIC number of the CRKP isolates for AMK were high (range between 1 and 16 μg/ml) but the strains were sensitive except 1 CRKP isolate that had a MIC of 16,384 μg/ml (Tables 1, 2). We interpreted the susceptibility of AMK were: S (sensitive), ≤16 μg/ml; I (intermediate), 32 μg/ml; R (resistant), ≥64 μg/ml. The 50% minimum inhibitory concentrations (MIC_50_) of COL, MEM and AMK were found to be 8, 16, and 4 μg/ml, respectively. The 90% minimum inhibitory concentrations (MIC_90_) of COL, MEM and AMK were 16, 128, and 8 μg/ml, respectively (Table 2). To determine if the isolates display resistance to MEM and AMK, we performed disk diffusion assays. We found that the resistant rates of the CRKP isolates to MEM and AMK were 100 and 2.5%, respectively.

**Table 2:** Antimicrobial resistance rates and MIC distributions of CRKP isolates.

	**MIC (μg/ml)**			
	**Cutoff value of resistance (%)**	**Range**	**MIC50**	**MIC90**
COL	>2 (92.5%)	2~32	8	16
MEM	≥4 (100%)	4~256	16	128
AMK	≥16 (97.5%)	1~16384	4	8

*MIC, minimum inhibitory concentration; AMK, amikacin; MIC50, MIC at which 50% of the isolates tested are inhibited; MIC90, MIC at which 90% of the isolates tested are inhibited; COL, colistin; MEM, meropenem*.

### Synergistic Effects of COL-MEM and COL-AMK Combinations

To determine whether treatment of CRKP isolates with a combination of either COL-MEM or COL-AMK exerts synergistic and additive effects, we performed the checkerboard experiments. We observed that FICI of the COL-MEM and COL-AMK combination was 0.484 ± 0.190 and 0.494 ± 0.269, respectively. Treatment of CRKP isolates with a combination of COL-MEM exhibited 65.0% synergistic and 35.0% additive effects while a combined treatment of COL-AMK resulted in 65.0% synergistic and 3.25% additive effects on 39 CRKP isolates tested (Table 1). The FICI value of 1 CRKP isolate was unable to be calculated because the high concentration of AMK did not meet the drug requirements of the combination (Table 1). It appeared that the treatment of COL-MEM showed better synergistic and additive effects than COL-AMK combination (*P* < 0.05). As expected, the combination of COL-MEM had better synergistic and additive effects on the COL-susceptible isolates than the COL-resistant isolates (*P* < 0.05; Table 3). In addition, the treatments of CRKP isolates with combinations of COL-MEM and COL-AMK decreased MICs of COL by 5.75- and 5.33-fold, respectively as compared to MIC of COL with COL treatment alone.

**Table 3:** Combination against colistin-susceptible and colistin-resistant CRKP isolates.

**Comb**	**COL-S**			**COL-R**			**Impact**	***p*-value**
	**Nb**	**Mean**	**SD**	**Nb**	**Mean**	**SD**		
COL+MEM	3	0.542	0.144	37	0.48	0.194	Synergistic+ Additive	<0.001
COL+AMK	3	0.875	0.572	36	0.462	0.212	Synergistic+ Additive	0.619
*p*-value	0.454			0.064				

*Comb, Combination; COL-S, colistin-susceptible; COL-R, colistin-resistant; COL, colistin; MEM, meropenem; AMK, amikacin*.

**Figure 1:**
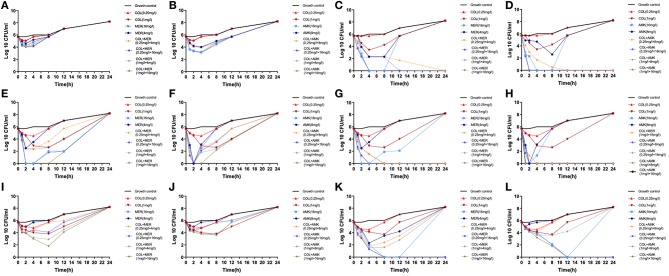
*In vitro* time-kill assays. Six CRKP isolates were treated with serum levels of colistin (COL), meropenem (MEM), or amikacin (AMK) alone or in a combination of COL MEM with either MEM or AMK. **(A)** CRKP26 isolate was treated with COL or MEM alone or in a combination of COL with MEM at the doses indicated. **(B)** CRKP26 isolate was treated with COL or AMK alone or in a com alone or a combination of COL with AMK at the doses indicated. **(C)** CRKP32 isolate was treated with COL or MEM alone or in a combination of COL with MEM at the doses indicated. **(D)** CRKP32 isolate was treated with COL or AMK alone or in a com alone or a combination of COL with AMK at the doses indicated. **(E)** CRKP34 isolate was treated with COL or MEM alone or in a combination of COL with MEM at the doses indicated. **(F)** CRKP34 isolate was treated with COL or AMK alone or in a com alone or a combination of COL with AMK at the doses indicated. **(G)** CRKP35 isolate was treated with COL or MEM alone or in a combination of COL with MEM at the doses indicated. **(H)** CRKP35 isolate was treated with COL or AMK alone or in a com alone or a combination of COL with AMK at the doses indicated. **(I)** CRKP36 isolate was treated with COL or MEM alone or in a combination of COL with MEM at the doses indicated. **(J)** CRKP36 isolate was treated with COL or AMK alone or in a com alone or a combination of COL with AMK at the doses indicated. **(K)** CRKP40 isolate was treated with COL or MEM alone or in a combination of COL with MEM at the doses indicated. **(L)** CRKP40 isolate was treated with COL or AMK alone or in a com alone or a combination of COL with AMK at the doses indicated.

We found that the monotherapies exhibited considerable regrowth as determined by the time-kill assay (Figure 1). Treatment of CRKP 6 isolates (CRKP26, 32, 34, 35, 36, and 40) with COL at a dose of 0.25 mg/L did not kill the bacteria but exerted a bacteriostatic effect on them (Figure 1). Treatment of 6 CRKP isolates with COL at 1 mg/L displayed a bactericidal activity against 2 CRKP isolates (CRKP34 and 35) (Figures 1E–H). Treatment of 6 CRKP isolates with MEM at 4 mg/L exhibited a bacteriostatic effect on 2 out of (CRKP26 and 36) (Figures 1A,I) and bactericidal action on 4 others (CRKP32, 34, 35, and P40) (Figures 1C,E,G,K). Treatment of 6 CRKP isolate with AMK at 8 mg/L inhibited the growth of 3 CRKP isolates (CRKP26, 36 isolate and 40) (Figures 1B,J,L) and killed 3 others (CRKP32, 34 and 35) (Figures 1D,F,H). Treatment of 6 CRKP isolates with MEM or AMK at 16 mg/L killed 4 of them (CRKP32, 34, 35, and 40) (Figures 1C–H,K,L) and suppressed the growth of 2 others (CRKP26 and 36) (Figures 1A,B,I,J). Treatment of CRKP32 and 35 isolates with COL combinations either with MEM or AMK exhibited synergic actions and completely killed the bacteria without regrowth while mono-treatment with either of them failed to show bactericidal effects (Figures 1C,D,G,H). These combinations included 0.25 mg/L COL and 4 mg/L MEM, 0.25 mg/L COL and 16 mg/L MEM, 1 mg/L COL and 4 mg/L MEM, 1 mg/L COL and 16 mg/L MEM, 0.25 mg/L COL and 8 mg/L AMK, 0.25 mg/L COL and 16 mg/L AMK, 1 mg/L COL and 8 mg/L AMK, and 1 mg/L COL and 16 mg/L AMK. Treatment of CRKP34, 36, and 40 isolates with a combination of 1 mg/L COL and 16 mg/L MEM initially inhibited their growth but prolonged treatment and culture resulted in bacterial regrowth (Figures 1E,I,K). While treatment with a combination of 1 mg/L COL and 16 mg/L AMK showed synergic effects on CRKP 34 and 40 isolates, continuation of culture in in the presence of antibiotics caused bacterial regrowth (Figures 1F,L). Similarly, treatments of CRKP34 isolate with a combination of 0.25 mg/L COL and 8 mg/L AMK, or a combination of 0.25 mg/L COL and 16 mg/L AMK displayed a synergy, but long term treatment resulted in bacterial regrowth (Figure 1F).

## Discussion

*Klebsiella pneumoniae* strains are usually harmless. They often live in human's intestines without giving any problems. But the bacteria can be very dangerous if they invade into other parts of the body when people are sick, with weak immune systems. The germs can become “superbugs” and they can cause pneumonia or spread through blood, resulting in serious problems. Treatment of *K. pneumoniae* infections usually starts with broad-spectrum antibiotics such as cephalosporins (cefotaxime and ceftriaxone), β-lactams and β-lactamase inhibitors, and carbapenems (imipenem or cilastatin). However, the dissemination of CRKP strains has limited the therapeutic options. Thus, treatment with polymyxins such as colistin abandoned many years ago has been re-implicated as one of antibiotics of the last resort for CRKP infections. Recent studies reported that *K. pneumoniae* also became resistant to COL because of the prolonged or inappropriate treatments, and the monotherapy of colistin for CRKP infection caused some problems (Zhang et al., 2017; Huang et al., 2018). In our study, we found that the single antibiotic treatment of CRKP isolates from our hospital with exhibited considerable regrowth Treatment of CRKP isolates with COL at a dose of 250 μg/L did not kill the bacteria but exerted a bacteriostatic effect on 6 CRKP isolates. Treatment of the bacteria with MEM at 4,000 μg/L exhibited a bacteriostatic effect on 3 CRKP isolates and treatment of the bacteria with AMK at 8,000 μg/L inhibited the growth of 3 CRKP isolates. Our observed phenotype of the CRKP isolates was consistent with what has recently been reported in the United states, indicating the hetero-resistance of CRKP isolates to the drugs contributes to bacteriostatic effect and regrowth (Band et al., 2018, 2019). Therefore, combination therapy is needed not only to fight the multiple-drug resistant CRKP isolates but also to prevent the emergence of the resistance during the treatment.

Antibacterial action generally falls into four mechanisms, three of which involve the inhibition or regulation of enzymes involved in cell wall biosynthesis, nucleic acid synthesis and DNA repair, or protein synthesis, respectively (Kapoor et al., 2017; Reygaert, 2018). The fourth mechanism involves the disruption of membrane structure (Farkas et al., 2017; Rempe et al., 2017). In other hand, the bacteria have become smarter and along with the antibiotics, massive imprudent usage of antibiotics in clinical practice has caused resistance of bacteria to antimicrobial agents. The biochemical resistance mechanisms used by bacteria include antibiotic inactivation, target modification, altered permeability, and “bypass” of metabolic pathway. Colistin is a cationic antimicrobial peptide that targets bacterial lipopolysaccharide (LPS), causing cell membrane leakage (Hancock, 1997; Kwa et al., 2007; Yu et al., 2015). Previous studies have shown that the increased modification of LPS by the *pmrHFIJKLM* operon, and the two-component systems PhoPQ and PmrAB with connector PmrD changed the negative charge and reduced susceptibility to colistin in *Enterobacteriaceae*, therefore contributing to colistin resistance (Gunn et al., 2000; McPhee et al., 2006; Yan et al., 2007). Recent studies also demonstrated that the insertion, amino acid changes, or deletion of *mgrB* relieved the inhibition of PhoQ phosphorylation, leading to increased expression of *pmrHFIJKLM* transcript and reduced susceptibility to colistin in *K. pneumonieae* (Cannatelli et al., 2014; Olaitan et al., 2014; Cheng et al., 2015). In addition, the plasmid-mediated COL resistance gene, MCR-1, was discovered in 2015 (Liu et al., 2016).

Meropenem inhibits bacterial cell wall synthesis and is highly resistant to degradation by β-lactamases or cephalosporinases while the primary mechanism of amikacin action is to bind to bacterial 30S ribosomal subunits and interferes with mRNA binding and tRNA acceptor sites, interfering with bacterial growth by disrupting normal protein synthesis and producing non-functional or toxic peptides. Because the reduction in colistin permeability is the major mechanism of KP resistance to colistin, we hypothesized that COL combination with MEM or AMK would sensitize each other to inhibit biofilm formation and increase the membrane permeability by targeting multiple proteins via distinct mechanisms, therefore exerting synergistic effects on CRKP isolates (Kim et al., 2015; Su et al., 2019). As expected, our studies showed that COL-AMK had obvious synergistic and additive effects, and antibiotic's MICs in the combination were significantly lower than those of monotherapies. Though the COL combinations with AMK and MEM had the same antibacterial activity against the CRKP isolates of both COL-S and COL-R, the synergistic effect of COL-AMK was superior to the additive effect while the synergistic and additive effects of COL-MEM on the strains were same. Our data indicated that treatment of CRKP isolates with COL combination with aminoglycoside class antimicrobials resulted in a better synergistic effect than did the COL-carbapenems combination. In addition, we demonstrated that treatments of NDM-producing CRKP isolates with COL combinations lead to a better efficacy of antibacterial activity than KPC-producing CRKP strains.

In this study, we applied both the checkerboard techniques and the time-kill assays for the evaluation of the efficacies of the antibiotics *in vitro* and obtained a great agreement from these independent experiments. The COL combinations had synergistic or additive effects on all CRKP isolates in the checkerboard technique except one on which a higher AMK concentration was needed to obtain the same effect. The COL combinations were significantly synergistic against all distinct ST CRKP isolates except the ST76 in the time-kill assay. The difference in the outcomes of experiments was because the mean value for steady-state concentrations of non-protein-bound drugs in humans did not meet the single-drug MIC *in vitro*. It appears the results of the time-kill assay were more reliable and safer. In our study, we observed regrowth of some strains in the presence of antibiotic combinations. It is possible that the resistant subpopulations were selected through antimicrobial pressure in the time-kill assay (Gupta et al., 2014). Alternatively, the CRKP strains might adapt environmental stimuli by altering the outer membrane and became resistant to the combination after the prolonged treatment *in vitro* (Meletis et al., 2011).

## Conclusion

In conclusion, we demonstrated that AMK was a better antibacterial agent against all CRKP isolates than MEM and COL *in vitro*. Treatments of 6 CRKP isolates at 16 μg/ml MEM or AMK in addition to 2 μg/ml COL completely killed 4 of them and suppressed the growth of 2 others (ST76 and ST11) while monotherapy showed no bactericidal effects. Our data suggest that COL could be a valid therapeutic option against COL-resistant CRKP isolates when used in combination with MEM or AMK. There are a few limitations in this study. First, while our *in vitro* experiments showed antimicrobial activity of two antibiotic combinations, and the experiments yielded statistical significances in the checkerboard technique and time-kill assays, the findings from this study need to be validated *in vivo* and in clinical trials. The isolates tested in the time kill assay should be further studies with the E-test for Colistin, Amikacin and Meropenem to confirm if the isolates exhibit phenotypic signs of hetero-resistance. Second, we only tested 6 CRKP isolates from a Healthcare Center with combination treatments. More tests are needed to determine if the combination treatment of two antibiotics is effective to other 34 CRKP isolates that have not been tested in this study as well as the CRKP isolates from other medical hospitals. Third, a pharmacokinetic evaluation of the antibiotics is also required to optimize the antibiotic combination regimens.

## Data Availability Statement

All datasets generated for this study are included in the article/supplementary material.

## Author Contributions

XZ conceived and designed the study. LY and JSZ wrote this paper and contributed equally to this work. LY, JSZ, YF, and YZ performed the experiments. YW, JZ, YG, and CL analyzed the data.

### Conflict of Interest

The authors declare that the research was conducted in the absence of any commercial or financial relationships that could be construed as a potential conflict of interest.
